# Carcinome epidermoide de l'appareil ungueal de l'Hallux sans atteinte osseuse: une observation chez un patient traité par amputation

**DOI:** 10.11604/pamj.2016.24.111.9166

**Published:** 2016-06-01

**Authors:** Koffi Léopold Krah, Mohamed Béchir Karray, Mouadh Nefiss, Christian Darga, Mohamed Bouabdellah, Mondher Kooli

**Affiliations:** 1 Service Orthopédie Traumatologie Hôpital Charles Nicolle, Tunis, Tunisie

**Keywords:** Appareil unguéal, carcinome épidermoïde, orteil, traitement chirurgical, Nail apparatus, squamous cell carcinoma, toe, surgical treatment

## Abstract

Le diagnostic tardif du carcinome épidermoïde de l'hallux a nécessité une amputation trans phalangienne proximale chez un patient âgée pris en charge en seconde main. C'est une lésion rare aux orteils qui peut mettre en jeu le pronostic fonctionnel du pied. Nous rapportons ce cas clinique dans le but de mettre en évidence les difficultés du diagnostic et les bases de l'amputation avec une revue de la littérature.

## Abstract (en)

The late diagnosis of squamous cell carcinoma of the hallux in an aged patient on second-line therapy led to a trans-proximal phalanx amputation. This is a rare lesion affecting the toes which may compromise the functional prognosis of the foot. This clinical case report with review of the literature aims to highlight the difficulties of diagnosis and indications for amputation.

## Introduction

Le carcinome épidermoïde est la plus fréquente des tumeurs de l'appareil unguéal [1, 2]. Il est plus observé aux doigts et rare aux orteils [2–4]. Sa localisation sur le gros orteil pose des problèmes diagnostics avec plusieurs lésions dont les mycosiques ou la tuberculose. Il est souvent méconnu ce qui réduit son incidence. Notre cas clinique évolue depuis deux ans chez une personne âgée avec des soins initiaux inutiles.

## Patient et observation

**Figure 1:**
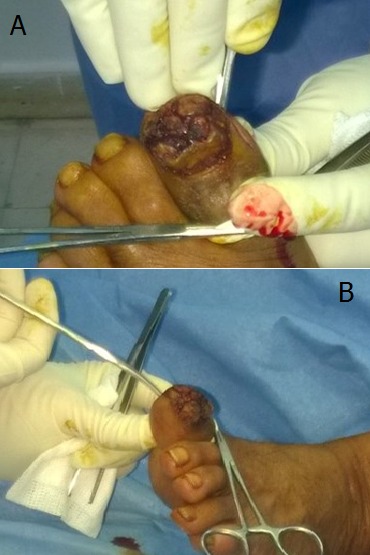
A) aspect tumoral sur une vue dorsale du pied; B) aspect de la tumeur sur une vue latéro-plantaire

**Figure 2:**
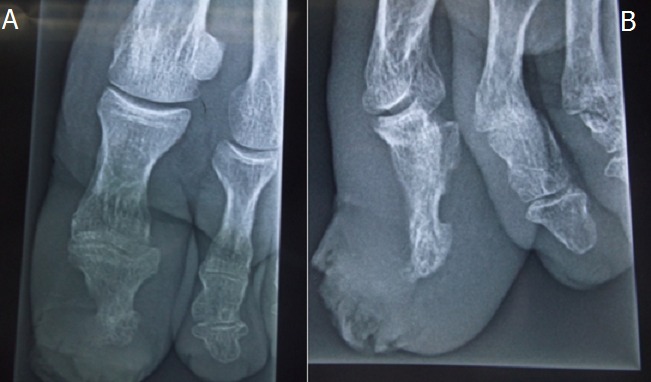
A) radiographie de face de l'orteil; B) radiographie de profil de l'orteil

**Figure 3:**
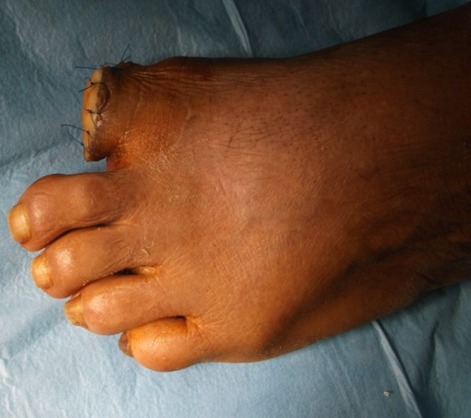
Aspect du moignon de l'orteil en post opératoire immédiat

**Figure 4:**
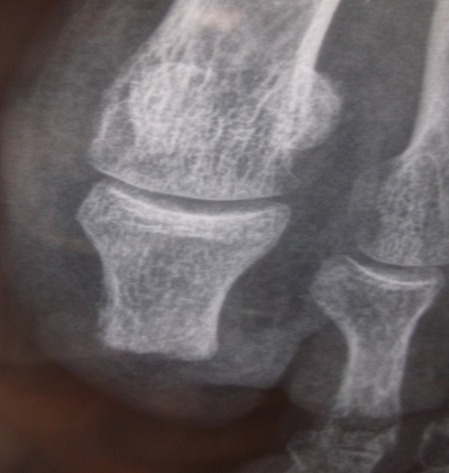
Radiographie de contrôle du moignon d'orteil en post opératoire au septième jour

Mr A.H, 75 ans, agriculteur, tabagique, sans antécédent pathologique notable a consulté en dermatologie pour une verruqueuse sous unguéale de l'hallux gauche qui a été traitée comme lésion mycosique sans succès dans un dispensaire. La maladie évoluait depuis deux ans. Une biopsie infructueuse a été faite puis une seconde un mois plus tard qui a permis de mettre en évidence une lésion épidermique papillomateuse et verruqueuse sans signe franc de malignité; toute fois un carcinome verruqueux n’était pas éliminé. Une exérèse à minima a été faite sans succès. La tumeur augmentait de taille avec surinfection locale réalisant une tumeur bourgeonnante kératosique verruqueuse sur la totalité du lit unguéal avec décollement de l'ongle (Figure 1). Elle débordait sur la peau environnante non pigmentée et était sensible avec des signes infectieux locaux. La radiographie de l'orteil (Figure 2) montrait des ostéophytes de la houppe phalangienne. Après avis d'un orthopédiste, une troisième biopsie a été faite qui a objectivé un carcinome épidermoïde bien différencié de type verruqueux sans atteinte osseuse. Les autres orteils et les doigts de la main étaient sans particularité. Le pied était de type égyptien sans vice architectural de l'avant-pied. Les aires ganglionnaires étaient libres. La radiographie des poumons était normale. Il a été réalisé une amputation trans phalangienne proximale de l'orteil (Figure 3, Figure 4), 6 mois après la première biopsie avec des suites hospitalières simples.

## Discussion

Le carcinome épidermoïde de l'orteil est rare [2–4]. Les cas publiés concernent le plus souvent une observation [5, 6] ou trois observations [7]. Il représente 2% dans l'ensemble des 58 cas de carcinome de l'appareil unguéal de l’étude de Lecerf [2]. Le problème qu'il pose est celui du diagnostic d'une tumeur de l'hallux. La présence d'ostéophytes pourrait faire évoquer un ostéome ostéoïde. L'exposition répétée aux pesticides liée à la profession agricole associée probablement aux traumatismes de l'orteil peut favoriser l'apparition de la tumeur dans notre cas. Les papillomavirus humains oncogènes en particulier le type 16 sont les facteurs de risque important de carcinome épidermoïde unguéal [4]. Aucun examen biologique n'a été fait dans ce sens dans notre cas. Dans les 3 cas des orteils de l’étude de Nasca la recherche par réaction de polymérisation en chaine de papillomavirus humain était négative [7]. Il serait peu probable de l'observer aux orteils [7]. L'homme est le plus atteint avec un âge souvent supérieur à 60 ans. L'atteinte est en général mono dactylique. Le délai de consultation est long (2 ans dans notre cas, 6 ans chez Lecerf). La forme verruqueuse est observée et les présentations cliniques sont souvent trompeuses (onychomycose puis verrue dans notre cas) avec des traitements locaux souvent inutiles [2, 5, 7, 8]. Le traitement antifungique réalisé dans le dispensaire peut faire penser à une contamination fungique de maladie de Bowen cutanée [9]. Malgré l'absence d'invasion osseuse et d'adénopathie, une amputation a été réalisée contrairement aux indications de la chirurgie de Mohs [10]. Le niveau d'amputation a été guidé par l'absence d'atteinte osseuse à l'anatomie pathologique et l’état local nécrotique en regard de la dernière phalange qui ne permettait pas une désarticulation inter phalangienne. L'articulation métatarso-phalangienne est importante dans le déroulement du pas. L'amputation liée à une extension ganglionnaire inguinale, a été associée à un curage dans l'un des cas de l’étude de Nasca [7]. Les métastases ont été observées 3 ans après l'amputation dans l’étude de Huang [5]. Kelly ne l'a pas observé après 2 ans [6].

## Conclusion

L'erreur du diagnostic liée à la méconnaissance de la pathologie dans le dispensaire puis l'insuffisance des techniques de biopsie et leur lecture ont favorisé l'extension de la tumeur. Le traitement est essentiellement chirurgical mais l'amputation dans notre cas est un échec de la prise en charge. La maladie est rare aux orteils peut-être parce qu'elle serait sous estimée.

## References

[1] Mikhail GR (1984). Subungual epidermoid carcinoma. J Am Acad Dermatol..

[2] Lecerf P, Richet B, Theunis A, Andre J (2013). A retrospective study of squamous cell carcinoma of the nail unit diagnosed in Belgian general hospital over a 15-year period. Journal of the American Academy of Dermatology..

[3] Tosti A, Richert B, Pazzaglia M, Scher RK, Daniel CR (2007). Tumeurs de l'appareil unguéal. Onychologie: diagnostic, traitement, chirurgie.

[4] Riddel C, Rashid R, Thomas V (2011). Ungual and periungual human papillomavirus associated squamous cell carcinoma: A review. J Am Acad Dermatol.

[5] Huang KC, Wen-Vei Hsu R, Lee KF, Li YY (2005). Late inguinal metastasis of a weil-differentiated subungual squamous cell carcinoma after radical toe amputation. Dermatologic Surgery..

[6] Kelly KJ, Kalani AD, Storrs S, Montenegro G, Fan C, Lee MH, Wallack MK (2008). Subungual squamous cell carcinoma of the toe: working toward a standardized therapeutic approach. Journal of surgical education..

[7] Nasca MR, Innocenzi D, Milali G (2004). Subungual squamous cel carcinoma of the toe: report of three cases. Dermatologic surgery..

[8] Hojyo-Tomoka MT, Chanssot-Deprez C, Vega-Memije ME, Dominguez-Chreif J (2006). Subungual squamous cell carcinoma of the first toe. International journal of dermatology..

[9] Dalle S, Depape L, Phan A, Balme B, Ronger-Savle S, Thomas L (2007). Squamous cell carcinoma of the nail apparatus: clinicopathological study of 35 cases. Br J Dermatol..

[10] Mohs FS (1941). Chemosurgery, a microscopically controled method of cancer excision. Arch Surg..

